# Transboundary aerosol transport process and its impact on aerosol-radiation-cloud feedbacks in springtime over Northeast Asia

**DOI:** 10.1038/s41598-022-08854-1

**Published:** 2022-03-22

**Authors:** Hyo-Jung Lee, Yu-Jin Jo, Seungwoo Kim, Daecheol Kim, Jong-Min Kim, Daniel Choi, Hyun-Young Jo, Juseon Bak, Shin-Young Park, Wonbae Jeon, Cheol-Hee Kim

**Affiliations:** 1 Institute of Environmental Research, Pusan National University, Busan, 46241 South Korea; 2 Department of Atmospheric Sciences, Pusan National University, Busan, 46241 South Korea; 3 Korea Science Academy of Korea Advanced Institute of Science and Technology, Busan, 47162 South Korea; 4 National Center for Fine Dust Information, Ministry of Environment, Cheongju, 28166 South Korea; 5 Korea Institute of Atmospheric Prediction System (KIAPS), Seoul, 07071 South Korea

**Keywords:** Climate sciences, Environmental sciences

## Abstract

Northeast Asia has been suffering from dramatic increases of particulate matter (PM) since the late 1990s, and it still continues to undergo haze despite various abating regulations. In this study, we investigated aerosol-cloud-precipitation (ACP) interactions with the varied PM, and the impact of long-range transport (LRT) process on ACP in springtime was assessed in Northeast Asia. Our long-term (1995–2019) analysis of PM_10_ exhibited the correlation with decreases of both sunshine duration and drizzle occurrences that can be interpreted as direct and indirect aerosol effects, while cloud cover induced by the varied PM_10_ was found only in more than 90% cloud cover (9/10–10/10 category). The online WRF-Chem with wind-blown dust simulation indicated that cloud water was affected by secondary inorganic aerosol (SIA) formation near the surface in upwind areas dominantly, whereas, along the LRT pathway, cloud water perturbation altitudes were increased quasi-linearly toward downward between 1 and 3 km. The gas-to-particle conversion ratios of sulfur ([SO_4_^2−^]/[SO_2_ + SO_4_^2−^]) and nitrogen ([NO_3_^−^]/[NO_2_ + NO_3_^−^]) both remain aloft long at the same vertical levels of most perturbed cloud altitude enough to be transported over long distance in springtime. Formations of sulfate and nitrate showed different ACP interaction timing; distinctive shifts in the ratios observed at the exit (Shanghai-Yellow Sea) by nitrate, and entrance areas (Seoul-Tokyo) by sulfate along the LRT pathway, respectively, with higher ratios of 0.8 or more in springtime. Our results indicate that ACP processes have been enhanced at a LRT-related altitude with different SIA production timings that can be considered in species-specific springtime PM abatements over Northeast Asia.

## Introduction

Northeast Asia is one of the fastest developing regions worldwide, receiving significant air pollution from various sources^1–3^. Although fine particulate matter (PM) levels have decreased significantly in recent years due to emission reduction policies, PM levels remain high with frequent and severe haze episodes in regions, especially during the spring period^4–6^. In addition to natural and anthropogenic emissions, PM_2.5_ levels in the atmosphere are affected by complex chemical transformations, including secondary formations and wet/dry deposition processes. Correspondingly, Northeast Asia is an area of increasing scientific interest for investigating aerosol-cloud-precipitation (ACP) feedbacks due to the complex aerosol-meteorology interactions occurring in this region.

Aerosols are affected directly and indirectly through ACP feedbacks. Previous studies indicate that aerosol direct effects are mainly affected by meteorological variables such as temperature and precipitation^7–10^, whereas aerosol indirect effects can promote reduced cloud drop sizes by lowering the collision efficiency, thereby affecting coalescence and collision processes^11,12^. Several changes in ACP feedbacks occur owing to these effects, including the suppression of weak rainfall (or drizzle)^13,14^, delaying precipitation time^15–17^, intensifying deep convective clouds^13,16^, and the upsurging in the surface rainfall^18^ over time. ACP interactions have been recognized to be complicated and exhibit regional and temporal variability uncertainties^16,17,19–22^, depending on the regional variations in aerosol loadings in the atmosphere.

Northeast Asia is an area where many studies have investigated the long-range transport (LRT) of air pollutants and the impact of transboundary transport of Chinese pollution on neighbouring countries through modelling and intensive measurements^23–26^. Anthropogenic aerosols, natural dust, ozone and its precursors, and carbon monoxide emitted from upwind areas, such as industrial areas in China, can be subjected to LRT to neighbouring downwind areas, including the western United States^27–37^. They showed that the LRT of pollutants plays an important role in modulating atmospheric composition and air quality. Thus, assessing the impacts of pollutant LRT on ACP interactions is becoming important in Northeast Asia.

**Figure 1:**
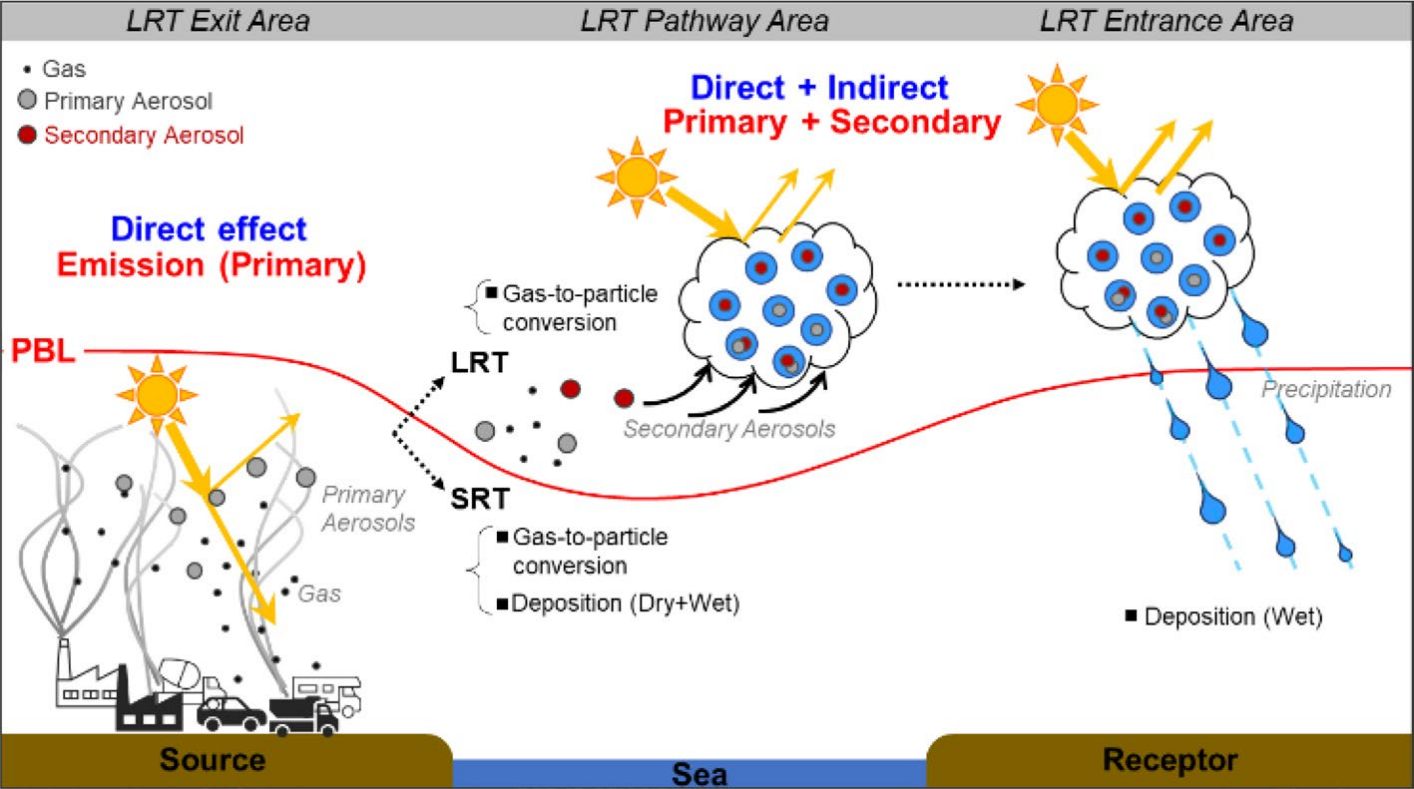
Schematic illustration of aerosol-cloud-precipitation interactions during the long-range transport process.

Secondary inorganic aerosol (SIA) and organic aerosol formations associated with the role of LRT on ACP interactions, have been rarely studied over Northeast Asia. Some studies have focused on horizontal transport pathways^37–40^ and vertical mixing structures^31,33,41–43^ over the belt area spanning the Yellow Sea in China and the Korean Peninsula. However, most regional-scale studies on ACP feedbacks have been conducted in the summer when precipitation rates are high^1,44–47^. Furthermore, few studies have investigated ACP feedbacks in an atmosphere with high aerosol loads (i.e., winter-spring season). For example, aerosol loadings in the spring are on average higher than in the summer at the surface and higher altitudes over Northeast Asia. The high-pressure systems that are accompanied by westerly and northwesterly winds move eastward frequently, and resulting ACP interactions occur in downwind areas affected by both primary aerosols near sources and secondary aerosols along the LRT pathway in Northeast Asia (Fig. 1).

Increased SIA-related emissions, including fine particulate matter and gas phase precursors (e.g., SO_2_ and NO_x_), originating from upwind source regions can modify cloud microphysics effectively when they are generated and subsequently move toward receptor regions. However, as quantitative measurements in the upper atmosphere are not available, in-situ measurements, satellite data, and numerous numerical experiments can be used to assess the impact of the LRT of pollutants on ACP interactions.

In the current measurement-simulation study, we first investigated the 25-year variation in cloud-precipitation properties: cloud cover, precipitation intensity, radiation, and sunshine duration, particularly in association with aerosol loadings: measured PM_10_. Next, an online WRF-Chem model including dust simulation was also applied to interpret the interactive ACP feedback process by diagnosing the direct and indirect aerosol effects on cloud invigoration and precipitation development during the LRT process. We carried out our study only in the spring season when the aerosol (PM_2.5_) concentrations are relatively higher and LRT processes occurred more frequently. During the analysis of ACP interactions, we explore how the LRT process and SIA can contribute to cloud precipitation and analyse their implications on regional weather.

## Results

### Long-term trends in meteorological factors and PM_10_

**Figure 2:**
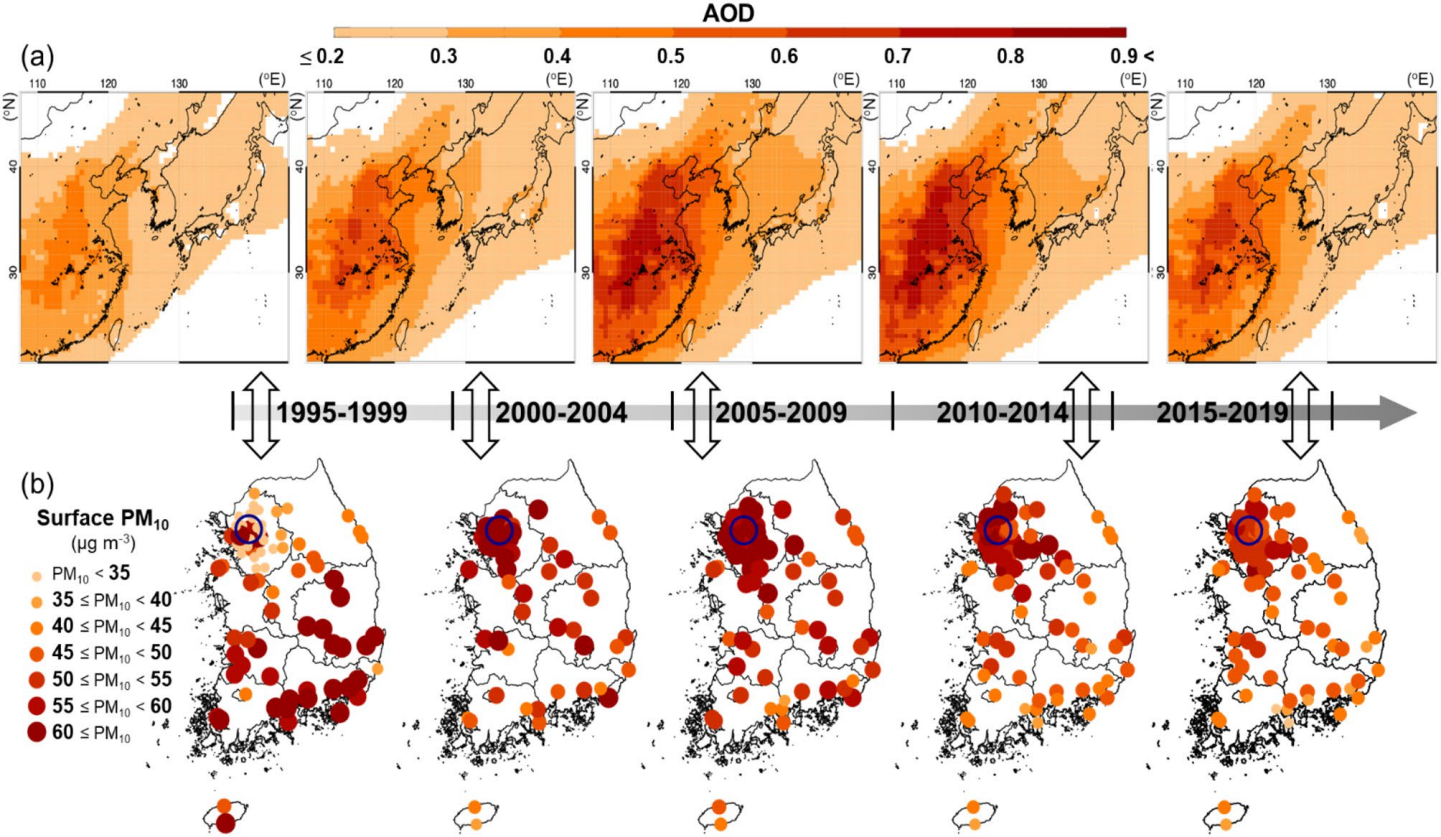
Time series of 5-year averaged horizontal distributions of (**a**) aerosol optical depth using MERRA-2 data in Northeast Asia and (**b**) in situ PM_10_ surface measurements in South Korea. The blue circles indicate the location of Seoul.

Figure 2 shows the long-term trends in the aerosol optical depth (AOD) over Northeast Asia, together with PM_10_ measurements in South Korea during 1995–2019. As a reference, the annual time series for PM_10_ concentrations in Seoul are also displayed in Figure S1. One thing to note is that there exist no adequate quantitative long-term aerosol (PM_2.5_) data, available PM_10_ measurements were used in this study for the long-term ACP interaction studies. In Fig. 2a, the spatiotemporal trends for AOD show significant increases over the period from 1995 to 2014, especially in urban and industrial areas near the Chinese inland (110–120° E, 25–40° N) such as Beijing-Tianjin-Hebei (BTH) in East China. However, in recent years (2015–2019), the AOD in East China was noticeably lower than the previous period, mainly due to the Atmospheric Pollution Prevention and Control Action Plan that has been strongly enforced since 2013 by the Chinese government^3,48^.

Figure 2b shows that PM_10_ concentrations in the Seoul metropolitan area (SMA) in Korea increased from 1995 to 2009; however, it decreased since the beginning of 2010 (Figure S1) owing to the implementation of the Special Air Quality Act in the Seoul metropolitan area by the Korean government in 2003 and 2010, which led to significant declines in PM_10_ concentrations over South Korea, including the SMA^36,49^. It is interesting to note in Figure S1 that the two starting years of abating policies (2003 and 2010) are consistent with the first and second most significant PM_10_ declining trends, respectively (Figure S1). Despite the recent decline in the AOD in Seoul and over Northeast Asia, the PM_10_ annual mean in Seoul remained high at 34 μg m^−3^ in 2019 compared to those of other metropolitan cities in 2019 such as Los Angeles (29 μg m^−3^), Tokyo (16 μg m^−3^), Paris (20 μg m^−3^), and London (18 μg m^−3^), as shown in Figure S1(a).

**Figure 3:**
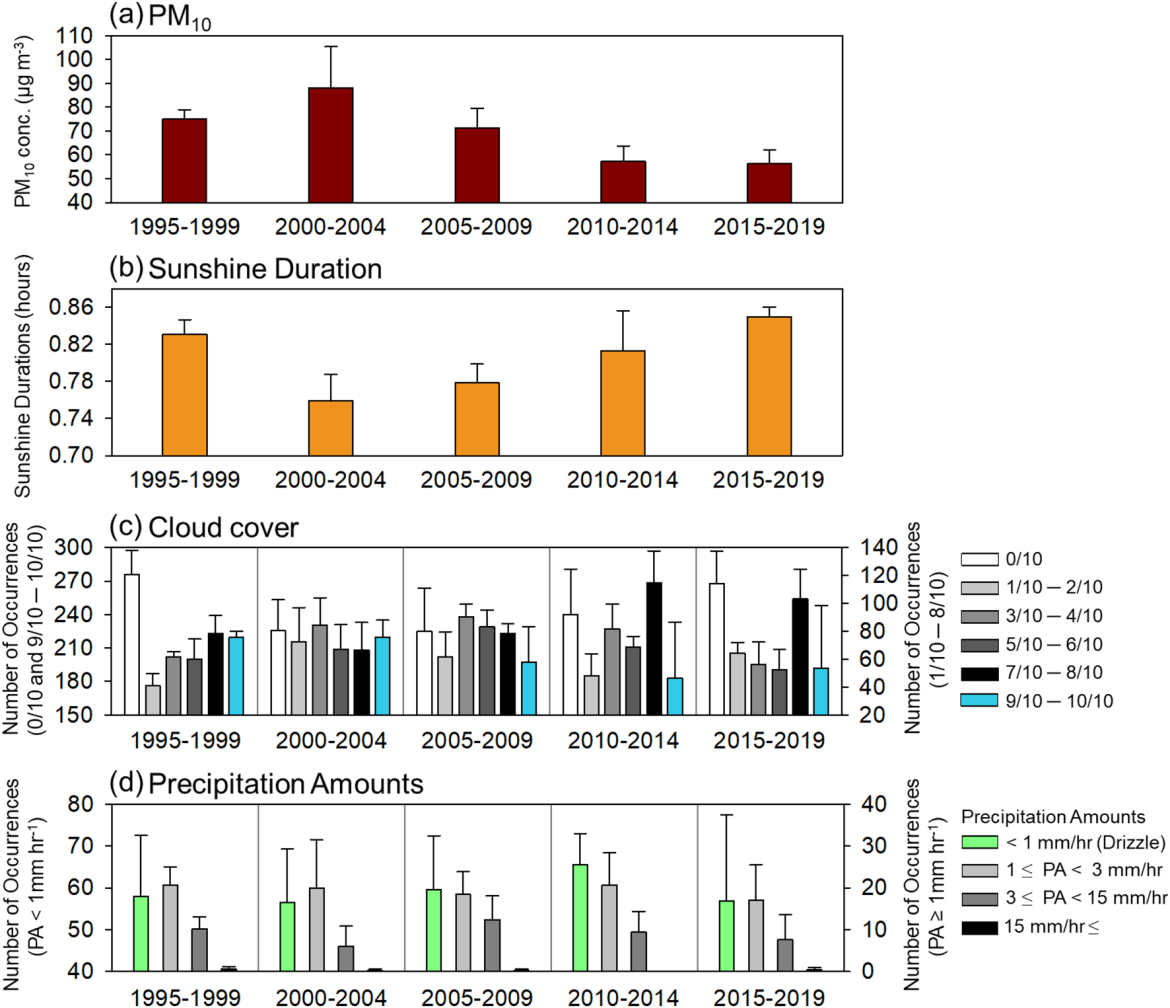
Long-term (1995–2019) springtime trends for (**a**) PM_10_ concentration, (**b**) sunshine duration, (**c**) the frequency of cloud cover for each cloud cover interval, and (**d**) the occurrences of precipitation intensities divided by hourly precipitation amounts in Seoul, South Korea.

The observed trends for sunshine duration (SD), cloud cover (CC), drizzle occurrences (DO), and precipitation amounts (PA) related to inter-annual AOD-PM_10_ changes provide insights into aerosol direct and indirect effects in Seoul. We defined in this work the drizzle as precipitation with its amount of < 1 mm hr^−1^, conformed to that in World Meteorological Organization (1975)^50^. Figure 3 shows that the SD decreased significantly during the period when PM_10_ concentrations increased from 2000 to 2002, and then gradually increased when the PM_10_ concentrations decreased from 2005 to the present (Figure S1, Fig. 3a), thereby indicating a strong inverse correlation between PM_10_ and SD in Seoul with the correlation coefficient (*r*) of − 0.73. This observation can be simply explained by the theory of direct and indirect aerosol effects, where the cloud formation process affected by aerosols dimmed incoming solar radiation and increased the cloud lifetime.

A similar relationship was found between PM_10_ and DO (Fig. 3d). For instance, DO tended to decrease when PM_10_ concentrations increased from 2000 to 2002, and it increased in 2003 when the PM_10_ reduction policy began. However, the relationship between PM_10_ and PA is not clear in our study, as there are more complex characteristics associated with PA relative to the other variables. The relationship between PM_10_ vs. CC showed a positive correlation for the 9/10–10/10 category, whereas other CC categories showed no obvious correlations. The occurrence of CC in the 9/10–10/10 category maintained higher during relatively higher PM_10_ period (2000–2002), and decreased since 2005 (Fig. 3c); these results are broadly consistent with the timing of increased AOD and PM_10_ over Northeastern Asia and in Seoul. We hypothesised that if the second aerosol indirect effect increases the lifetime of clouds, then changes in the occurrence of clouds that concurrently suppress precipitation should be limited to low-level non-precipitating clouds with precipitation just beginning to form.

It is challenging to establish a robust relationship between ACP processes and long-term observations of CC and PM_10_ in highly polluted areas; this is because other influential factors, such as climatological factors and large-scale meteorological circulation, contribute to the ACP process at larger and broader scales. In Seoul, aerosol levels have been affected by both local emissions and the LRT process, during which aerosols can be removed by dry or wet deposition at near surface, while SIA can be generated in the atmosphere aloft. As Fig. 1 indicated, aerosol precursors originating from the upwind areas of Seoul (i.e., urban and industrial regions in China) formed SIA (and other secondary aerosols) during LRT, and that SIA-induced ACP feedbacks can newly occur as they move toward receptor regions. Although there are no available chemical or physical measurement data for higher altitudes at which clouds are present, potential ACP effects by LRT can be represented by online and offline WRF-Chem simulations for the LRT dominant cases.

### Aerosol-induced changes in radiation, cloud, and precipitation properties for the LRT cases

Our selection of LRT cases was based on the criteria described by Jo and Kim (2013)^51^, which distinguished LRT conditions by tracking consecutive 6-day synoptic weather charts and air trajectories. In our study, the LRT cases during the 2018 spring season occurred on 10–11 March 2018, 23–24 March 2018, and 19 April 2018. In Figure S2, both model—MERRA2 reanalysis data, and model—surface PM_2.5_ measurements, are compared for chosen periods, showing on-line model’s good capability in capturing the increasing and decreasing trends for PM_2.5_ concentrations in Seoul. The index of agreement was 0.73 (Figure S2a) and the correlation coefficient (*r*) of the spatial AOD distributions was also 0.67 in Northeast Asia (Figure S2b), thereby corroborating the representation of the ACP interactions by the online model. As for the model validation of cloud-precipitation properties, the simulated shortwave radiation (vs. observed SD), cloud water (vs. observed CC), and precipitation amounts (vs. observed PA) were compared against observations (Figure S3). Note that the variable in the modeling and measurement does not match. The online WRF-Chem model produced acceptable simulations for SD and CC and captured long-term trends, facilitating interpretations of PM_2.5_–cloud interactions.

**Figure 4:**
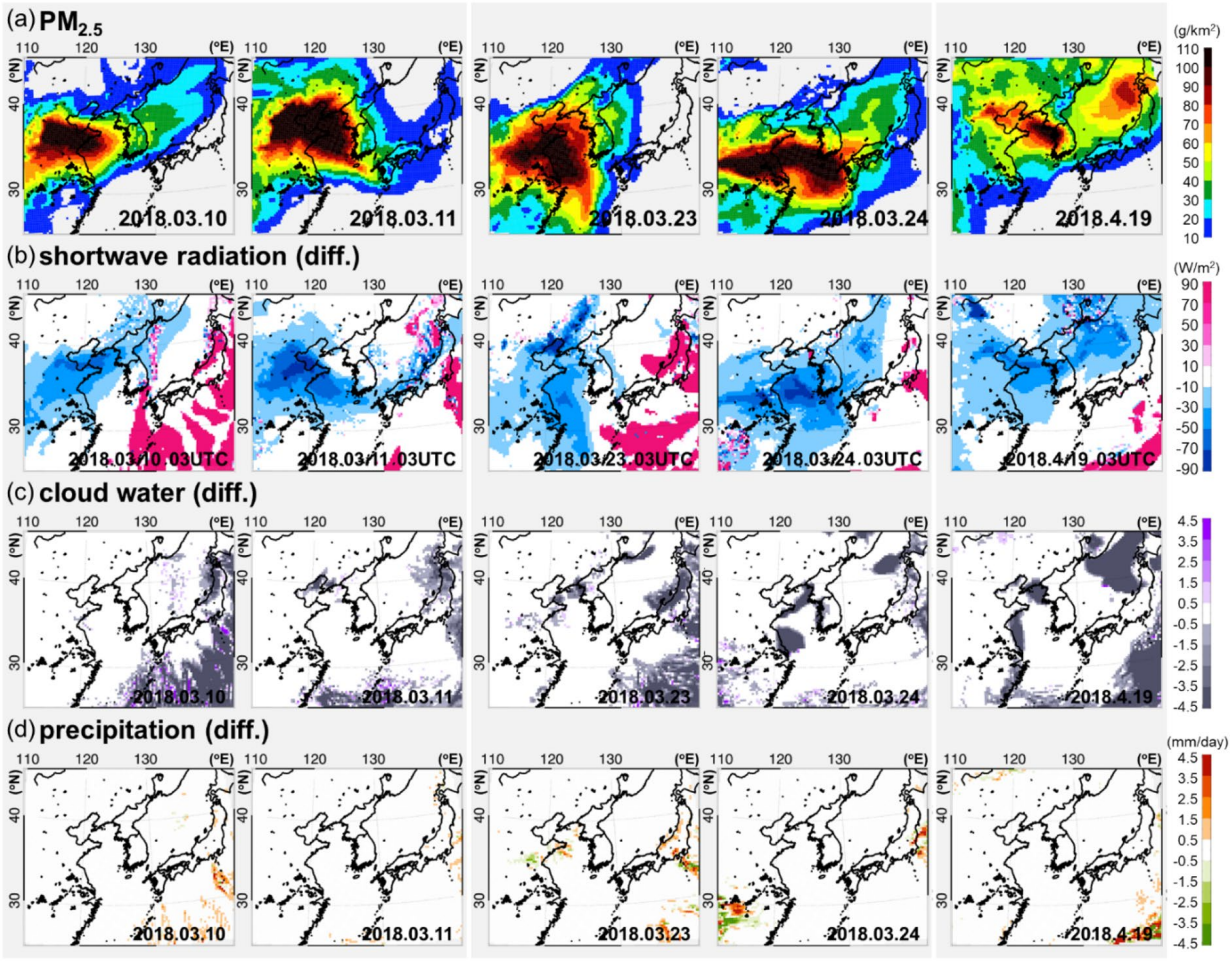
(**a**) Horizontal distributions of vertically integrated PM_2.5_ column concentrations during selected high PM_2.5_ episodes from March to April 2018. The impact of aerosol-induced differences between online and offline modelling results for (**b**) shortwave radiation at 0300 UTC, (**c**) daily cloud water mixing ratio vertically integrated from the surface to 50 hPa (top of model vertical layers), and (**d**) daily accumulated precipitation amounts for 10, 11, 23, and 24 March 2018 and 19 April 2018.

Figure 4 shows the online simulated PM_2.5_ and ACP variables such as shortwave radiation, cloud water, and precipitation with and without ACP interactions for chosen LRT-dominant periods. Figure 4b reveals the shortwave radiation differences (equivalent to the meaning of SD) near the BTH-Yellow Sea-SMA belt area on 24 March 2018 during the LRT process (i.e., areas with high PM_2.5_ concentrations). Shortwave radiation was perturbed by ACP interactions up to ~ 70 W m^−2^ lower (the maximum bias was found at ~ 12 KST local time), reflecting the aerosol direct effect (albedo changes) and the aerosol indirect effect (cloud cover increases). These shortwave radiation differences have driven up to ~ 10% in our study at higher PM_2.5_ concentrations along the belt area, during the LRT toward receptor regions (Figure S4).

There was also a more immediate change in cloud water from the aerosol-driven shortwave radiation differences (Fig. 4c) during the LRT process. The cloud water was lower along the high cloud water regions at the exit (i.e., coastal areas in East China) and entrance areas (i.e., coastal areas or the Yellow Sea ocean areas) of the LRT pathway. These results are consistent with those of previous studies on the high sensitivity of cloud water and CCN to relative humidity, as shown by Kang et al.^47^. Our results and those of Kang et al.^47^ show the overall decrease in cloud water by the aerosol effect under high humidity over the Yellow Sea, while other studies report opposite results^44,47^. A potential reason for this discrepancy is that only spring episodes were investigated in this study, while previous studies are based on summer season with higher precipitation condition and relatively lower aerosol loadings. In addition, these previous studies indicated that cloud water during the summer wet periods is increasing because of ACP interactions. It should be also noted that although there might be more local variations, ACP effects may produce different results depending on the cloud types such as non-precipitating low-level clouds and/or deep convective cumulus clouds^52^.

However, daily PA showed no significant differences (or a small increase) over Northeast Asia, including the belt area (Fig. 4d). This is because the high PM_2.5_ cases are not compatible with heavy precipitation days due to the wet removal process by precipitation; instead, we confirmed that aerosols can affect the enhancement or suppression of PA before and after the periods characterized by high PM_2.5_ concentrations by altering precipitation development in highly polluted areas (Figure S5). In particular, a small increase by 3.5 mm on average (mostly less than 10 mm) in daily PA was simulated over the non-LRT period during March–April over Southern China near Shanghai and the Yellow Sea (Figure S5), which is consistent with previous results^43^.

### Roles of aerosols in the vertical changes of clouds during LRT

**Figure 5:**
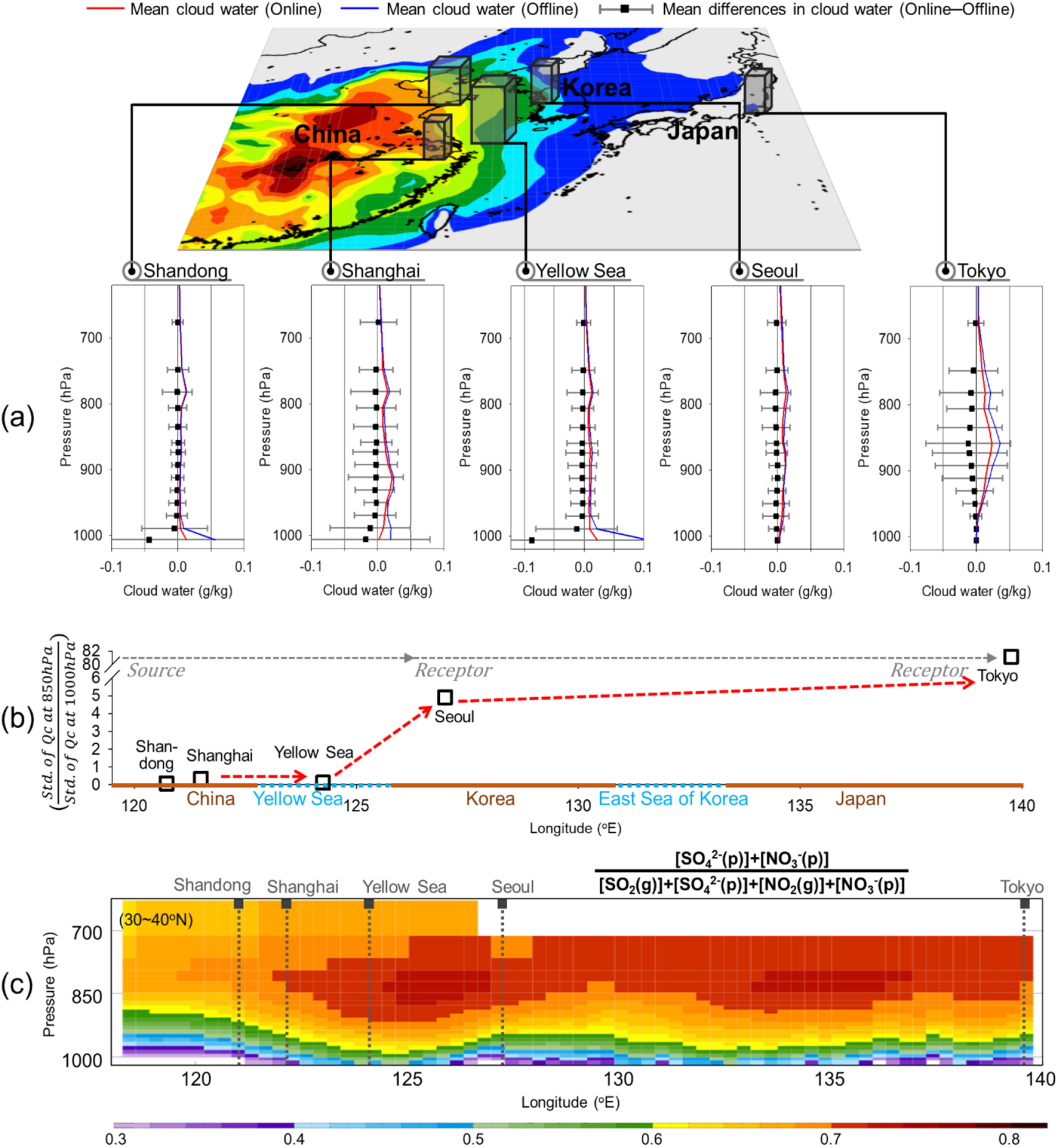
(**a**) Vertical profiles of online (red solid line) and offline (blue solid line) WRF-Chem-simulated cloud water mixing ratios from the ground to 600 hPa isobaric surface in Shandong, Shanghai, the Yellow Sea, Seoul, and Tokyo averaged from March to April 2018. The black squares and grey horizontal lines represent the mean and standard deviation of cloud water differences between online and offline WRF-Chem modelling results, respectively. (**b**) The ratio of standard deviations of cloud water differences between 850 and 1000 hPa in Shandong, Shanghai, the Yellow Sea, Seoul, and Tokyo. (**c**) Vertical distributions of gas-to-particle conversion ratios of sulfate and nitrate simulated by online WRF-Chem modelling. The separated sulfate and nitrate oxidation ratios are also illustrated in Figure S6.

Aerosols primarily can provide an intermediate stage for ACP interactions and have high potential to be enhanced by SIA formation during LRT pathways. These assumptions can be confirmed by online and offline numerical sensitivity experiments. We focused on the vertical profiles of cloud water perturbations from March to April 2018 during periods characterized by high PM_2.5_ concentrations in Shanghai, Shandong, Yellow Sea, Seoul, and Tokyo (Fig. 5).

The results showed that in Shandong and Shanghai, where PM_2.5_ concentrations were relatively high, the cloud water was largely reduced by aerosol interactions by almost 50% near the surface (i.e., < 1000 hPa isobaric altitude), while no differences were found at altitudes higher than 950 hPa (Fig. 5a). However, unlike upstream area, cloud water perturbations over Seoul and Tokyo were found at much higher altitudes, such as 950–700 hPa (Fig. 5a). The vertically averaged differences between 950 and 700 hPa are − 0.28, − 2.66, − 1.68, and − 7.08 μg kg^−1^ in Shandong, Shanghai, Seoul, and Tokyo, respectively. The estimated ratios of cloud water variance between the surface (1000 hPa) and 850 hPa (= σ_850hPa_/σ_1000 hPa_) were 0.1, 0.3, 0.1, 4.9, and 81 at Shandong, Shanghai, Yellow Sea, Seoul, and Tokyo, respectively, indicating a clear increase as the aerosol moved from west (exit area) to east (entrance area) (Fig. 5b). This trend strongly suggests that ACP interactions occurred during LRT, and it is worth paying special attention to the limited layers ranging from 900 to 700 hPa isobaric surface (equivalent to 1–3 km above the surface) are related to atmospheric boundary layers or residual layers in the LRT entrance areas over Northeast Asia.

Two chemical components were examined from gas-to-particle partitioning perspectives to confirm the role of SIA. Figure 5c and S6 show the vertical distributions of the sulfur oxidation ratio (SOR) (= [SO_4_^2−^]/[SO_2_ + SO_4_^2−^]) and nitrogen oxidation ratio (NOR) (= [NO_3_^−^]/[NO_2_ + NO_3_^−^]). The ratios both generally remain aloft long enough to be transported over long distance with high values of 0.8 or more over the Yellow Sea and Korean Peninsula. The distributions at altitudes of ~ 850 to 700 hPa (approximately 1.5–3 km) are consistent with significant cloud water perturbations, thereby corroborating the representation by the model. Here, it is also interesting to note the two distinctive significant jumps in the chemical composition ratios; (1) LRT exit area (Shanghai-Yellow Sea), and (2) LRT-entrance areas (Seoul-Tokyo). The former was identified as nitrate and the latter as sulfate (Figure S6).

## Discussion

The main goals of this study were to investigate the influence of aerosol effects on cloud-precipitation in association with LRT over Northeast Asia. We first analysed the observed cloud variables over a long-term period (1995–2019), and numerous numerical model simulations were performed to verify the observed characteristics. The results showed that in Seoul and South Korea, PM_10_ and SD were negatively correlated while PM_10_ and CC were positively correlated in the 9/10–10/10 cloud category. However, PM_10_ and PA did not show a significant relationship overall. However, increased PA (i.e., drizzle) was found in springtime in Northeast Asia, while other studies showed both increases in cloud water and suppression of rainfall during the strong precipitation period, such as the summer monsoon season. This implies that aerosol-precipitation may contain considerable levels of uncertainty, varying predominantly according to the moisture (humidity) levels (i.e., season), the vertical depth of convection, and local variation of non-precipitating low-level clouds by aerosol characteristics. These uncertainties can be traced to a number of assumptions made in more comprehensive model over the periods of monsoon vs. non-monsoon season to better quantify ACP sensitivities over Northeast Asia.

The cloud water affected by aerosols was typically found near the surface level in the LRT exit areas (Shandong and Shanghai) when PM_2.5_ plumes were moving; consequently, cloud water perturbations were observed at approximately 1–3 km above the surface over receptor regions in Northeast Asia. As dominant aerosol sources are mostly located at or near the surface, the aerosol properties decrease strongly with altitude. However, aerosol-led evolutions of ACP interactions are possible during the LRT process, especially by loading secondary generated aerosols. The online model simulation showed that ACP effects during LRT immediately reduced shortwave radiation along the plumes characterized by high PM_2.5_ concentrations up to ~ 10% in Northeast Asia. The cloud water perturbed by aerosols was found only near the surface level in the LRT exit areas (Shandong and Shanghai), and the cloud water was largely reduced at a 1000 hPa isobaric altitude or lower. Cloud water was often perturbed by aerosols at higher altitudes, particularly between 950 and 700 hPa, in downstream areas such as Seoul and Tokyo. The estimated ratios of the cloud water perturbations between two layers (1000 hPa and 850 hPa) (= σ_850 hPa_/σ_1000 hPa_) are 0.1, 0.3, 0.1, 4.9, and 81, at Shandong, Shanghai, Yellow Sea, Seoul, and Tokyo, respectively, showing quasi-linear increasing rate toward downward between 1000 hPa and 850 hPa.

The role of SIA was also examined. Sulfur and nitrogen oxidation ratios (= [SO_4_^2−^]/[SO_2_ + SO_4_^2−^], [NO_3_^−^ ]/[NO_2_ + NO_3_^−^]) both generally remain aloft long enough to be transported over long distance with high ratios of 0.8 over the Yellow Sea and Korean Peninsula at approximately 1.5–3 km; these results are consistent with the altitude associated with significant cloud water perturbation. Nitrate induced aerosol effects more actively over LRT exit area (Shanghai-the Yellow Sea), and by sulfate belatedly over LRT-entrance areas (Seoul-Tokyo). This is due to the differences in chemical characteristics between nitrogen and sulfur oxidation in the atmosphere, associated with its interaction with cloud water. An important oxidation process in sulfur chemistry is SO_2_ to sulfuric acid which has very low vapor pressure and remains in the particle phase, and oxidation process is taking place at large distances from the emission sources, except for the SO_2_ aqueous phase reaction process where more rapid reactions can occur. Thus, these processes depend on the extent to which SO_2_-H_2_O_2_-O_3_-cloud environments are activated^53,54^. Nitrogen oxidation processes are relatively more complicated, involving the O_2_-NO_x_-VOC cycle and N_2_O_5_ night-time heterogeneous reactions. Our modeling and measurement analysis of ACP effects were mainly focusing on secondary inorganic pollutants, particularly based on the high proportion of SIA components in PM_2.5_ species over Seoul^6^. In recent studies, however, the roles of primary aerosol components are also reported in association with LRT process. For example, the effects of primary aerosols (i.e., high BC) on meso-scale meteorological condition in North China Plain and Yangtze River delta in China^55^, and also their association with secondary aerosol formations via heterogeneous reaction process^56^ during the long-range transport episodes in China. Such results are indicating the importance of aerosol chemical compositions over the exit area of LRT process, and suggesting that the well-characterized components and its interaction via radiative processes are together the key factors within clouds. Nevertheless, when we limiting the domain to the Northeast Asia, the significant insights inferred from this study will give the reasons leading to the regulations of SIA-ACP interactions in the atmosphere.

The current study provides significant insights into the roles of the LRT process for ACP interactions and species-specific PM abatements. A more detailed study, including simultaneous and comprehensive, in-situ, aircraft, and remote sensing measurements, is needed to improve the understanding of ACP interactions over Northeast Asia. And the outcomes of aerosol effects from this study and related researches also need to be reflected to operational air quality and meso-scale weather forecasting for better prediction capabilities over Northeast Asia.

## Methods

### In situ observation

Long-term (1995–2019) trends for PM_10_ observed in South Korea were employed in this study. PM_10_ measurements were collected from the entire area of the Korean Peninsula by using the National Ambient Air Monitoring Information System (NAMIS) operated by the Korean Ministry of Environment (KMOE) (https://www.airkorea.or.kr/, last accessed on 23 September 2021). Additional PM_10_ measurements in Seoul (the capital city of South Korea), which is operated by Seoul Municipality (https://cleanair.seoul.go.kr/statistics/monthAverage, last access: 23 September 2021), were used for interpreting PM_10_ variations in Seoul. Surface PM_2.5_ data were used for model verification over the upwind area obtained from the China National Environmental Monitoring Center (CNEMC) available on its website (http://www.cnemc.cn, last access: 5 February 2022). We also used hourly measurements of sunshine duration (SD), cloud cover (CC), drizzle occurrences (DO), and precipitation amounts (PA) over Korea, which have been provided by the Korea Meteorological Administration (KMA, https://data.kma.go.kr/, last access: 24 June 2021), to analyse the long-term trends associated with the cloud observational data. Here, the CC data were measured semiquantitatively in 3-h intervals on a scale from 0/10 to 10/10, and the SD was measured on an hourly basis using a rotating-type EKO instrument with an internal calibration system at all stations. The records of hourly PA were divided into four categories of precipitation intensity following KMA guidelines^57^: PA < 1 mm hr^−1^ (drizzle), 1 mm hr^−1^ ≤ PA < 3 mm hr^−1^, 3 mm hr^−1^ ≤ PA < 15 mm hr^−1^, and 15 mm hr^−1^ ≤ PA. The observed sunshine duration (SD) was used as a proxy for atmospheric turbidity, which in turn was affected by aerosol-induced dimming and brightening phenomenon. As clouds and rainfall are mainly affected by the summer monsoon, we performed a model-measurement study focusing on the spring season (March–April–May) when the atmospheric aerosol concentration was the highest in Northeast Asia. The WRF-Chem evaluations are also made extensively by using the ‘hourly’ observed SD, CC, and PA that were all of great importance, from the ACP perspectives.

### Reanalysis data (MERRA-2)

We also employed satellite observations, the Modern-Era Retrospective Analysis for Research and Applications, Version 2 (MERRA-2), to estimate and evaluate spatiotemporal trends of simulated PM_2.5_ in Northeast Asia. MERRA-2 has a resolution of approximately 0.5° × 0.625° and 72 hybrid-eta levels from the surface to 0.1 hPa^55^. The reanalysis data is computed on a latitude–longitude grid at the same spatial resolution using a 3-D variation data assimilation (3DVAR) algorithm. The algorithm is based on the Gridpoint Statistical Interpolation (GSI) with a 6-h update cycle using the Goddard Earth Observing System Model, Version 5 (GEOS-5) with Atmospheric Data Assimilation System (ADAS), version 5.12.4^58,59^. We used the monthly mean aerosol optical depth (AOD) over the Northeast Asia domain since satellite data for PM_10_ was unavailable. The AOD is a parameter of the aerosol extinction coefficient integrated from the earth’s surface to the top of the atmosphere, representing the attenuation of solar radiation caused by aerosols^60^. The AOD reanalysis data were also used to indirectly verify the horizontal features of high PM_2.5_ plumes simulated by WRF-Chem.

### WRF-Chem model data

The WRF-Chem (ver.3.9.1) model adopted here is a regional scale online meteorology-chemistry model, namely: Weather Research and Forecasting (WRF) model coupled with chemistry modules^61,62^. In the WRF-Chem model, aerosol is acting as Cloud Condensation Nuclei (CCN), combined with the cloud microphysics, and allows interactive feedbacks via two processes: (1) affecting cloud droplet number and cloud radiative properties, and in turn (2) clouds alter aerosol size and composition via wet scavenging and aqueous processes^39^. Natural dust from Mongolia and western China was considered by employing improved dust scheme in WRF-Chem model. Details of WRF-Chem configuration together with employed dust scheme in this study were found in Supplementary file. The model simulations were carried out from 1 March to 30 April 2018, when occurrence frequency of high PM events was relatively high. The horizontal and vertical configurations over the WRF-Chem model domain (Figure S7) are as follows: (1) three nested domains with different horizontal grid spacings of 27 km (d01), 9 km (d02), and 3 km (d03); and (2) vertical structures with 29 layers in the terrain-following eta coordinate system. Meteorological initial boundary conditions were taken from the 6-hourly National Center for Environmental Prediction Final (NCEP/FNL) Operational Global Analysis data at a horizontal resolution of 1° × 1°. The physical parameters of the WRF-Chem model used in this study are listed in Table S1. Both online and offline simulations employed identical configurations including the horizontal and vertical grids, microphysics option, and meteorological initial boundary conditions over the modelling domain. In both simulations, a spin-up time of 5 days (1–5 March 2018) was applied. The characteristics of ACP processes during the LRT of pollutants were explored by inferring the differences between with (online) and without (offline) ACP interactions in two simulations. The online simulation considered aerosol-induced microphysics for vertically integrated PM_2.5_, shortwave radiation, vertically integrated cloud water, and daily PA during the LRT cases with high PM_2.5_ concentrations over Northeast Asia.

### Graphics software

All contour plots were produced using Interactive Data Language (IDL, https://www.harrisgeospatial.com/Software-Technology/IDL). All bar charts and scatter plots were plotted using the Sigma Plot program (http://www.sigmaplot.co.uk/products/sigmaplot/sigmaplot-details.php).

## Supplementary Information

Supplementary Information.

## Data Availability

The MERRA-2 reanalysis data analysed during the current study are available at https://disc.sci.gsfc.nasa.gov. All measured data and WRF-Chem results related to this paper are available from the corresponding author on reasonable request.
