# Supplementary material for: Transboundary aerosol transport process and its impact on aerosol-radiation-cloud feedbacks in springtime over Northeast Asia
Source: Sci Rep. 2022 Mar 22;12:4870. doi: 10.1038/s41598-022-08854-1 (PMC8941152; doi:10.1038/s41598-022-08854-1)
Supplement: Supplementary file 1 — Supplementary Information. [file 41598_2022_8854_MOESM1_ESM.docx]

*Supplementary information:*

Transboundary Aerosol Transport Process and Its Impact on Aerosol-Radiation-Cloud Feedbacks in springtime over Northeast Asia

Hyo-Jung Lee^1^, Yu-Jin Jo^2^, Seungwoo Kim^3^, Daecheol Kim^3^, Jong-Min Kim^2^, Daniel Choi^4^, Hyun-Young Jo^2^, Juseon Bak^2^, Shin-Young Park^5^, Wonbae Jeon^2^, and Cheol-Hee Kim^1,2*^

^1^ Institute of Environmental Research, Pusan National University, Busan 46241, South Korea

^2^ Department of Atmospheric Sciences, Pusan National University, Busan 46241, South Korea

^3^ Korea Science Academy of Korea Advanced Institute of Science and Technology, Busan 47162, South Korea

^4^ National Center for Fine Dust Information, Ministry of Environment, Cheongju 28166, South Korea

^5^ Korea Institute of Atmospheric Prediction System (KIAPS), Seoul 07071, South Korea

*Corresponding author: [chkim2@pusan.ac.kr](mailto:chkim2@pusan.ac.kr)

**Supplementary Information:**

Details of WRF-Chem model configuration

Supplementary Table: 1

Supplementary Figure: 1-7

References

**Details of WRF-Chem model configuration**

In this study, the physical options of WRF-Chem model employed Lin-scheme microphysics option, YSU boundary layer physics^1^, the NOAH land surface model^2^, a Rapid Radiative Transfer Model (RRTM) longwave and Goddard shortwave radiation schemes^3^, and the Grell‐Freitas ensemble cumulus parameterization with radiative feedback and shallow convection^4^.

The Regional Atmospheric Chemistry (RACM)‐Earth System Research Laboratory^5^ and the Modal Aerosol Dynamics model for Europe/Secondary Organic Aerosol Model (MADE/SORGAM)^6,7^ chemistry schemes were adopted here as the gas and aerosol chemistry options in WRF-Chem model. Table S1 shows the more detailed gas and aerosol chemistry option. The selected aerosol scheme, MADE/SORGAM, includes comprehensive aqueous-phase chemistry including SO_4_^2-^ and NO_3_^-^ wet deposition. All of these chemistry mechanisms were compiled using the Kinetic Pre-processor method coupled with the WRF‐Chem model. As for dust scheme, the improved GOCART dust scheme^8-9^ coupled with the MADE/SORGAM aerosol scheme were employed in this study.

The anthropogenic emission data used in our WRF-Chem simulations were calculated from the KORUS-AQ (Korea-United States Air Quality) emission inventories (KORUS v.2)^10-13^. For biogenic emissions, Model of Emissions of Gases and Aerosols from Nature (MEGAN) v2.04 emission^14^ was adopted in our WRF-Chem simulation.

**Table S1. Configuration of Weather Research and Forecasting model coupled with Chemistry (WRF-Chem).**

| **WRF-Chem Version** | **3.9.1** |
| --- | --- |
| Horizontal Resolution | 27km, 9km, 3km |
| Vertical Layer | 29 |
| IC/BC Condition | UM global forecasting data (25km) |
| Microphysics | Lin et al. scheme |
| Longwave Radiation | Rapid Radiative Transfer Mode (RRTM) scheme |
| Shortwave Radiation | Goddard shortwave scheme |
| Cumulus Parameterization | Grell 3D Ensemble scheme^*^ |
| Planetary Boundary Layer | YSU scheme |
| Gas Chemistry | NOAA/ESRL RACM Chemistry |
| Aerosol Chemistry | MADE/SORGAM aerosol using KPP library |
| Biogenic Emission | MEGAN |
| Anthropogenic Emission | KORUS v.5 |

***^*^*** *Cumulus parameterization was not used for 3 km × 3 km resolution domain*


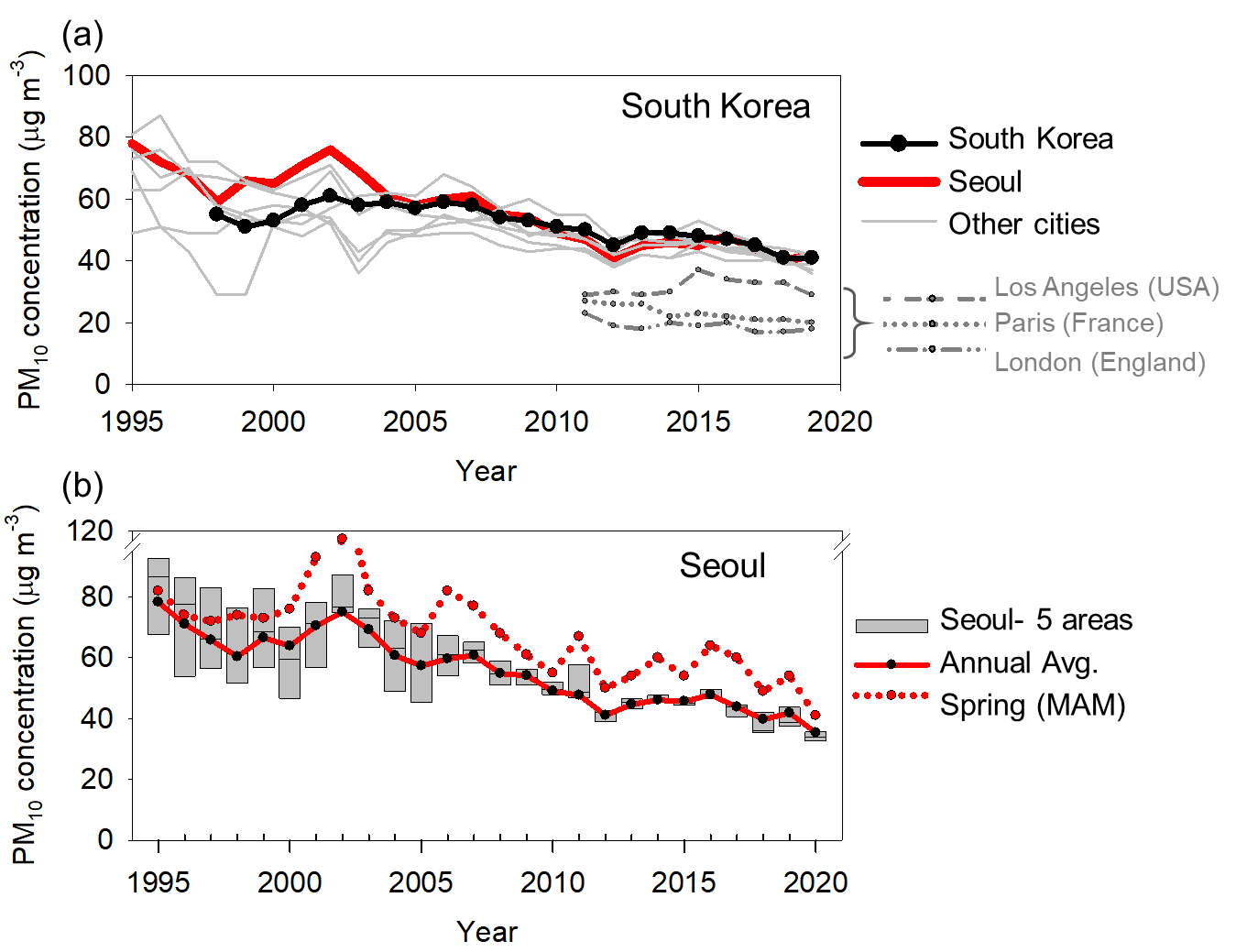


**Figure S1.** (a) Annual changes in PM_10_ measurements in South Korea for 1995─2019. Black line with black circle indicates PM_10_ annual means averaged for all NAMIS stations. The red line and gray lines, respectively, present annual PM_10_ trends in Seoul and other six urban areas; Busan, Daegu, Daejeon, Ulsan, Gwangju, and Incheon in South Korea. Gray dotted lines display PM_10_ mean values over the major cities in other countries. (b) Annual changes in monthly PM_10_ data provided from Seoul Air Quality Information website. Red line with black circle and red dotted line with red circle indicate the annual means of the whole seasons, and spring (March ─ April ─ May) season only, respectively. The gray box means the differences in PM_10_ annual mean in five districts in Seoul where long-term measurement dataset can be obtained in this analysis.


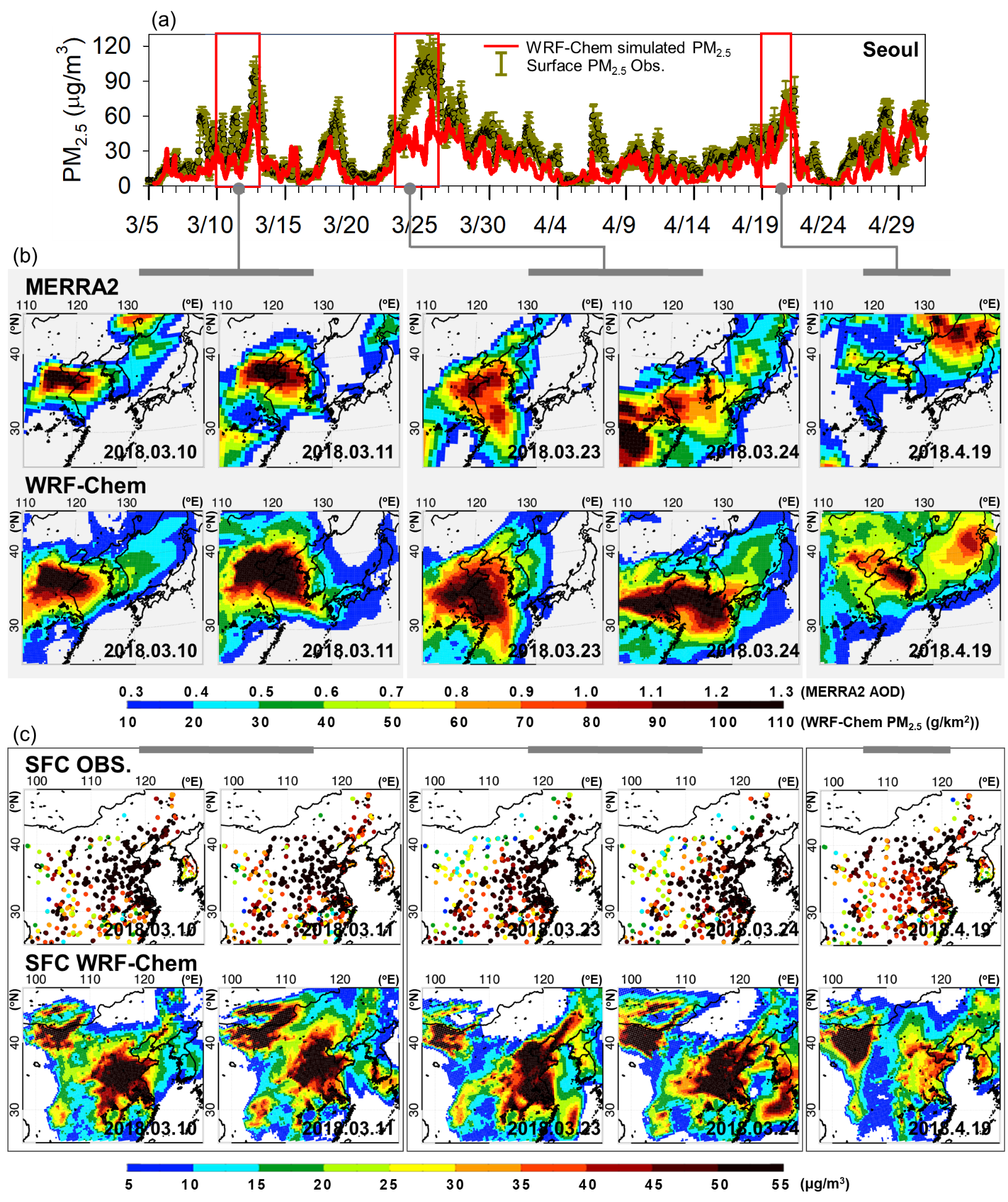


**Figure S2.** (a) Time series of hourly PM_2.5_ concentrations simulated by online WRF-Chem modeling (red line) and measurements from NAMIS stations (olive line). The vertical olive line indicates (mean)±(one standard deviation) of hourly PM_2.5_ concentration obtained from NAMIS stations in Seoul. (b) The spatial distributions of Aerosol Optical Depth (AOD) obtained from MERRA2 (top) and vertical integrated PM_2.5_ concentrations simulated by online WRF-Chem model (bottom) during the high PM_2.5_ episodes. (c) The spatial distributions of observed (top) and simulated (bottom) daily surface PM_2.5_ concentrations. Pattern correlation coefficient and index of agreement between obs. vs. model was 0.54, and 0.58, respectively, calculated from distributions shown in (c).


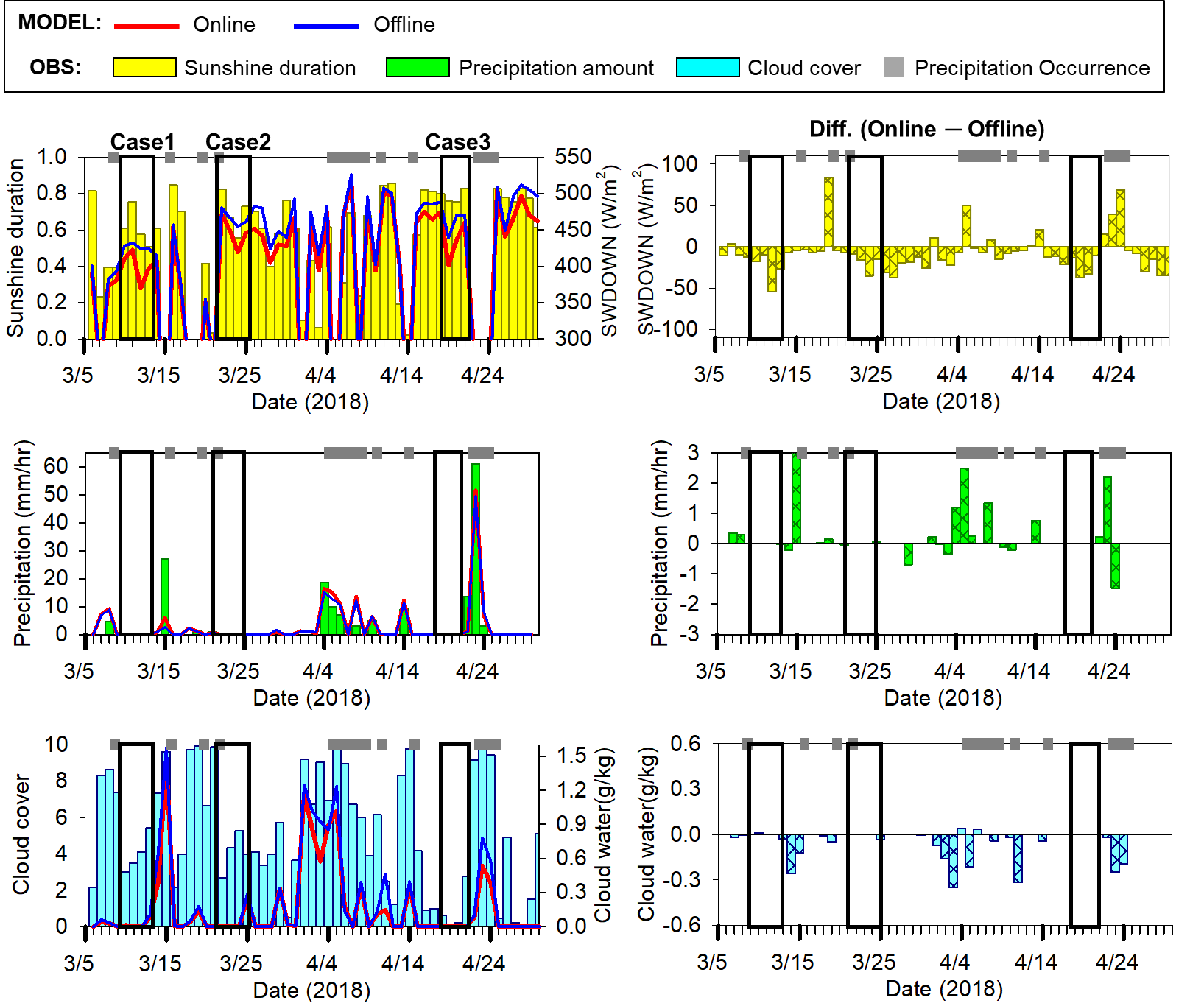


**Figure S3.** (Left panel) Time series of simulated shortwave radiation (SWDOWN), precipitation amount and cloud water simulated by online (red line) and offline (blue line) WRF-Chem. Observed sunshine duration (yellow bar), precipitation amount (green bar), and vertical integrated cloud cover (light blue bar) in meteorological observation site in Seoul. (Right panel) Time series of the differences between online and offline WRF-Chem model results for shortwave radiation, precipitation amount, and vertical integrated cloud water.


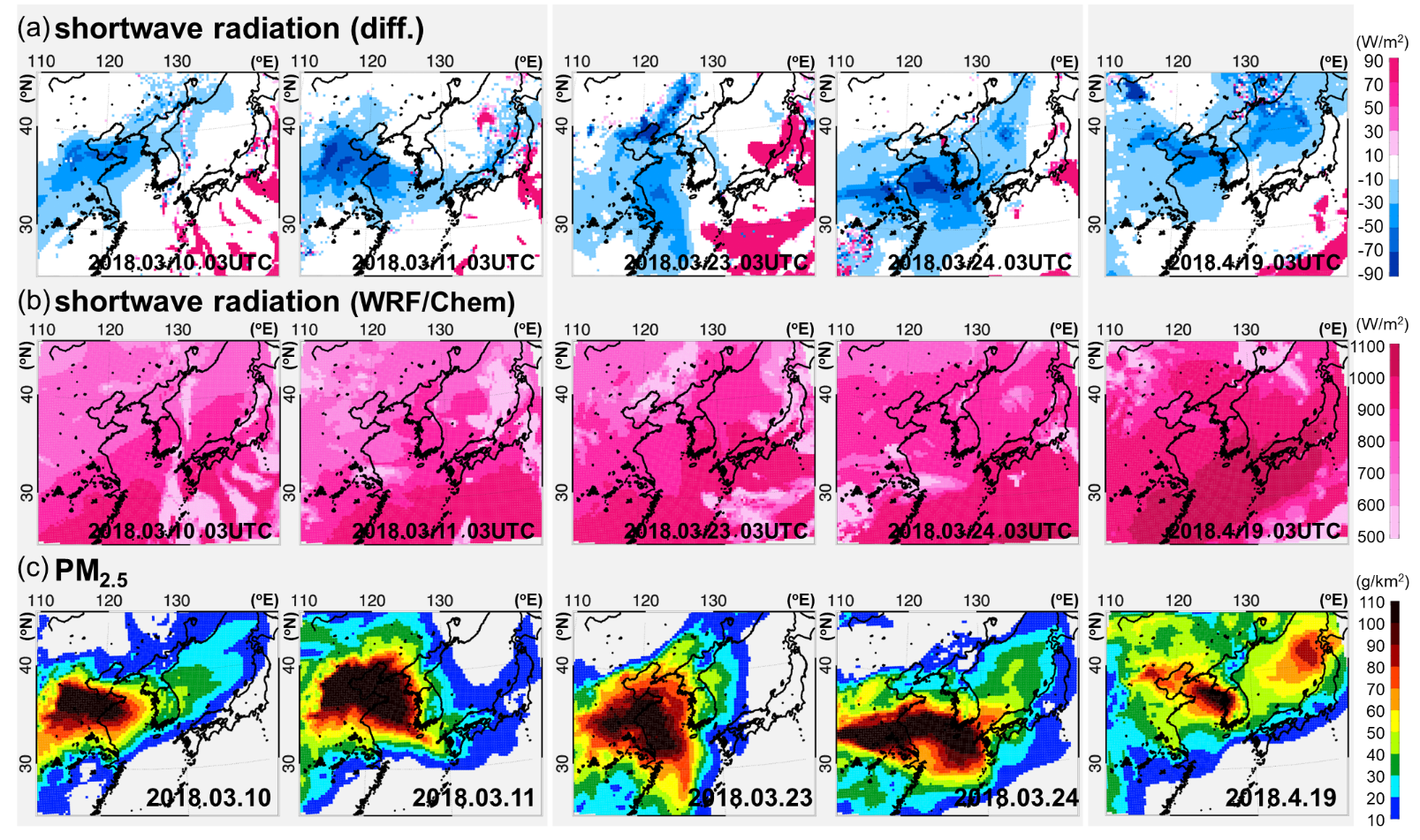


**Figure S4.** (a) Spatial distributions of shortwave radiation differences between online and offline WRF-Chem modeling during high PM_2.5_ episodes. (b) Spatial distributions of shortwave radiation simulated by online WRF-Chem modeling. (c) Spatial distributions of vertical integrated PM_2.5_ concentrations simulated by online WRF-Chem modeling.


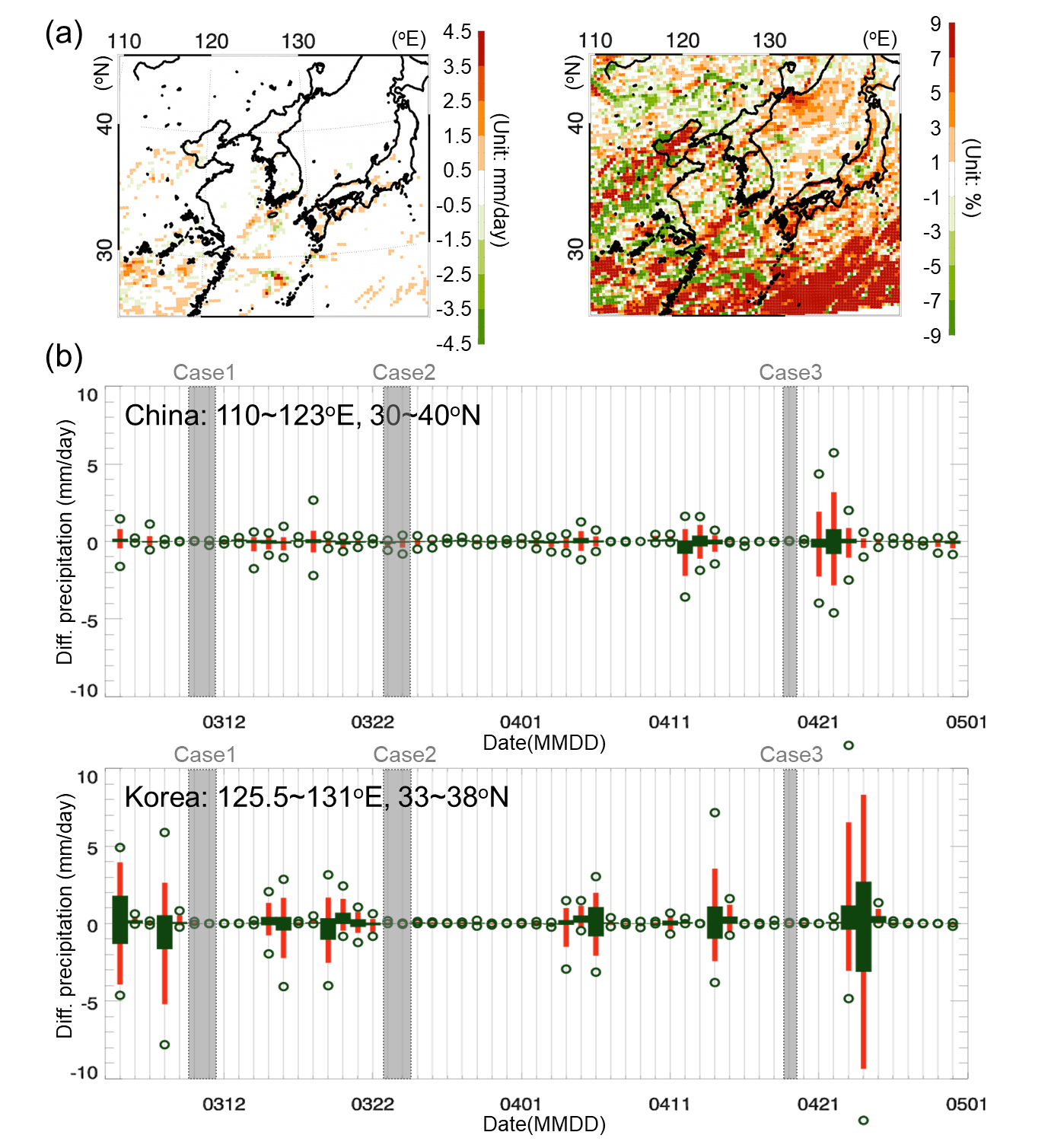


**Figure S5.** (a) The difference of daily precipitation amounts between online and offline WRF-Chem modeling results averaged for the precipitation cases (left), and precipitation difference (%) between two simulations (online and offline) normalized by online WRF-Chem simulations (right). (b) Time series of precipitation differences between online and offline WRF-Chem simulations for China and Korea. The green dots indicate 5^th^ and 95^th^ percentiles of precipitation differences. Red vertical bars and green vertical boxes show the ranges of the 10^th^─90^th^ percentile and the 25^th^─75^th^ percentiles, respectively.


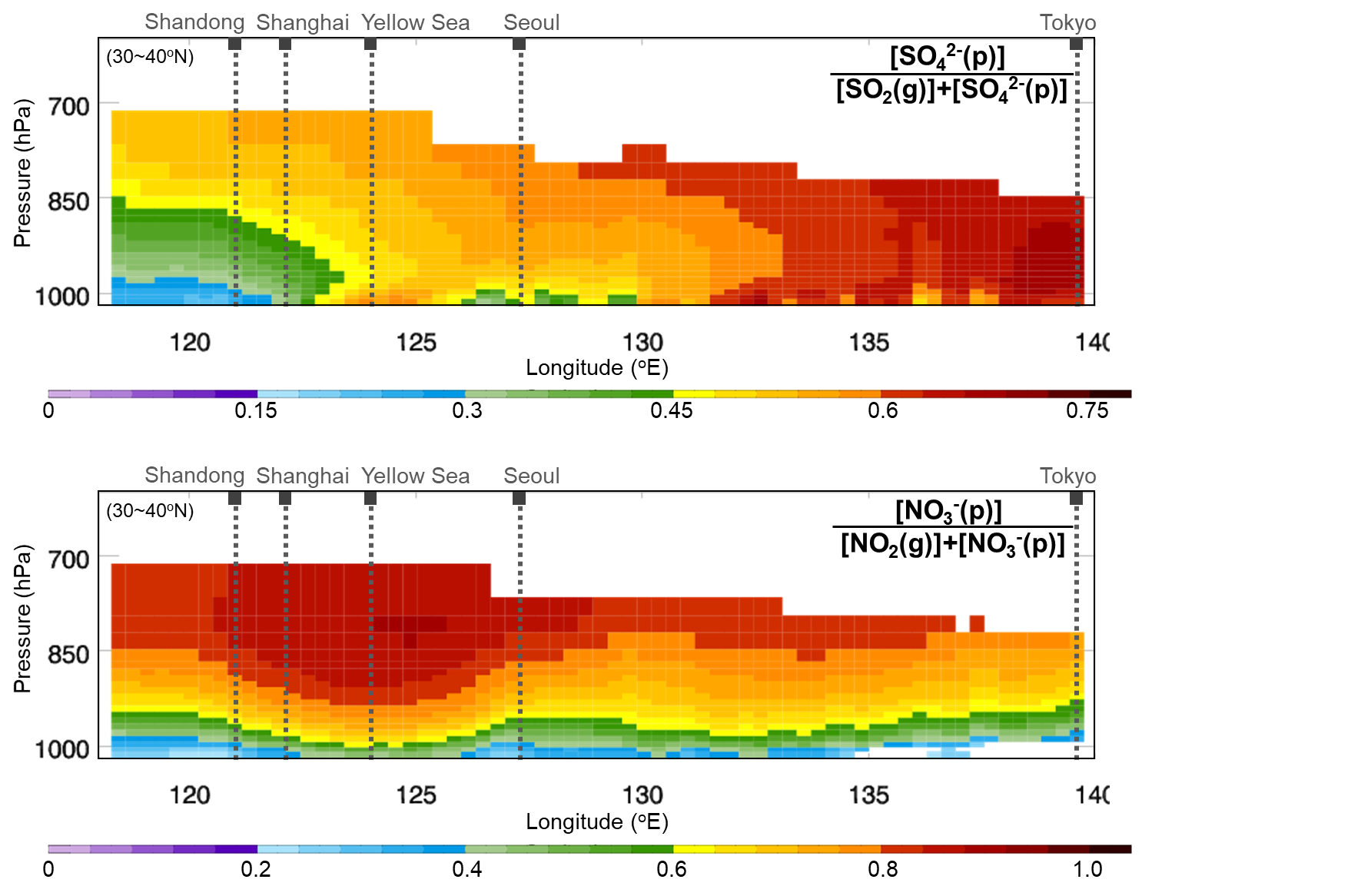


**Figure S6**. Vertical distributions of gas-to-particle conversion ratios of sulfate (top) and nitrate (bottom) simulated by online WRF-Chem modelling.


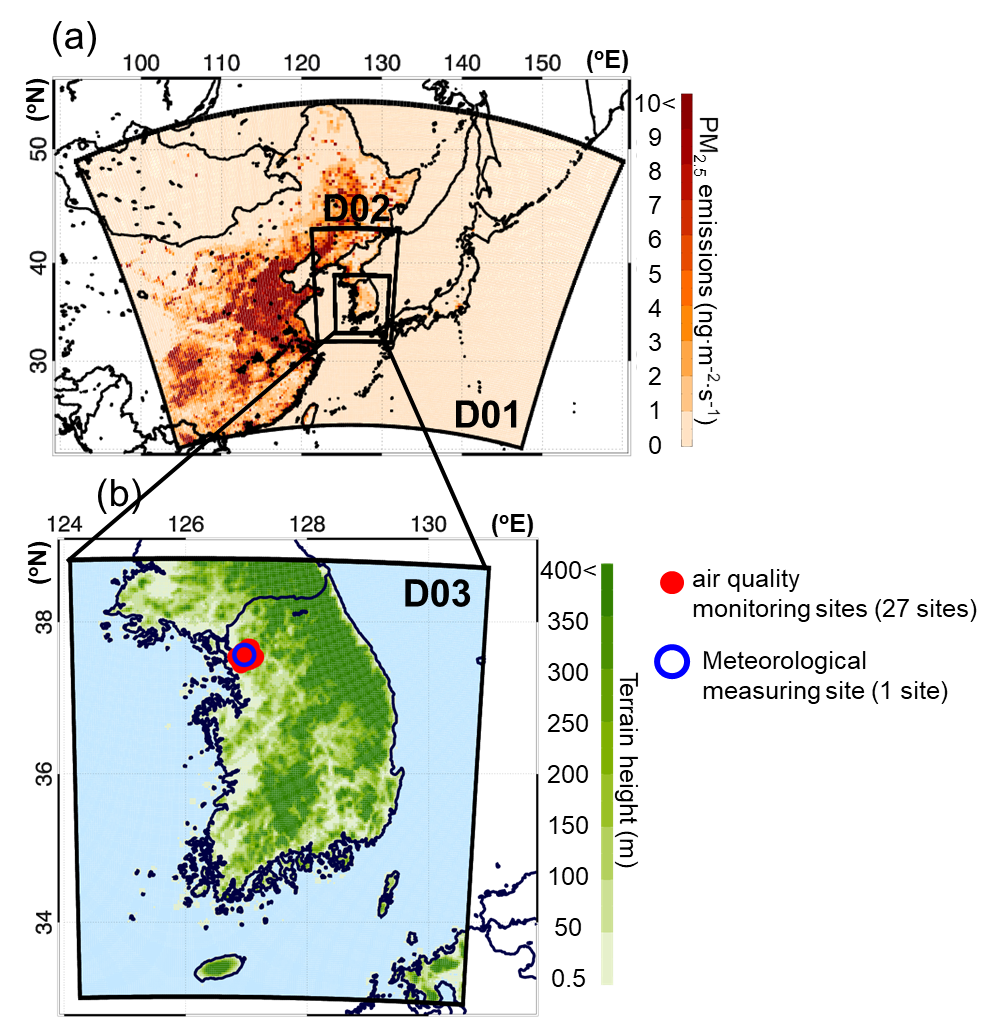


**Figure S7.** (a) Three nested domains with 27km, 9km, and 3km grid spacings used in WRF and WRF-Chem simulations. Here, primary PM_2.5_ emissions averaged was shown for the period of April-May 2018. (b) Third nested domain with the indication of observation locations of aerosol concentration (red circle) and meteorological observations (blue circle). Observation data were both employed to analyze long-term variations and model verifications.

**References**

1. Hong, S. Y., Noh, Y. and Dudhia, J. A new vertical diffusion package with an explicit treatment of entrainment processes. *Mon. Wea. Rev.* **134**, 2318–2341 (2006).

2. Chen, F. and Dudhia, J. Coupling and advanced land surface‐hydrology model with the Penn State‐NCAR MM5 modeling system. Part I: Model implementation and sensitivity. *Mon. Wea. Rev.***129**, 569–585 (2001).

3. Iacono, M. J., Delamere, J. S., Mlawer, E. J., Shephard, M. W., Clough, S. A. and Collins, W. D. Radiative forcing by long‐lived greenhouse gases: Calculations with the AER radiative transfer models. *J. Geophys. Res.* **113**, D13103 (2008).

4. Grell, G. A. and Freitas, S. A scale and aerosol aware stochastic convective parameterization for weather and air quality modeling. *Atmos. Chem. Phys.* **14**(10), 5233–5250 (2014).

5. Kim, S. W., Heckel, A., Frost, G. J., Richter, A., Gleason, J., Burrows, J. P. et al. NO_2_ columns in the western United States observed from space and simulated by a regional chemistry model and their implications for NO_x_ emissions, *J. Geophys. Res.* **114**, D11301 (2009).

6. Ackermann, I. J., Hass, H., Memmesheimer, M., Ebel, A., Binkowski, F. S. and Shankar, U. Modal aerosol dynamics model for Europe: Development and first applications. *Atmos. Environ.* **32**(17), 2981–2999 (1998).

7. Schell, B., Ackermann, I. J., Hass, H., Binkowski, F. S. and Ebel, A. Modeling the formation of secondary organic aerosol within a comprehensive air quality model system. *J. Geophys. Res.* **106**(D22), 28275–28293 (2001).

8. Zhao, C., Liu, X., Leung, L.R., Johnson, B., McFarlane, S.A., Gustafson, W.I., Jr., Fast, J.D., Easter, R. The spatial distribution of mineral dust and its shortwave radiative forcing over North Africa: Modeling sensitivities to dust emissions and aerosol size treatments. *Atmos. Chem. Phys.* **10**, 8821–8838 (2010)

9. Zhao, C., Chen, S., Leung, L.R., Qian, Y., Kok, J.F., Zaveri, R.A., Huang, J. Uncertainty in modeling dust mass balance and radiative forcing from size parameterization. *Atmos. Chem. Phys.* **13**, 10733–10753 (2013).

10. Woo, J. H., Choi, K. C., Kim, H. K., Baek, B. H., Jang, M., Eum, J. H., Song, C. H., Ma, Y. L., Sunwoo, Y., Chang, L. S. et al. Development of an anthropogenic emissions processing system for Asia using SMOKE. *Atmos. Environ.* **58**, 5–13 (2012).

11. Jang, Y., Lee, Y., Kim, J., Kim, Y., Woo, J. H. Improvement China Point Source for Improving Bottom-Up Emission Inventory. *Asia-Pac. J. Atmos. Sci.* (2019).

12. Oak, Y. J., Park, R. J., Schroeder, J. R., Crawford, J. H., Blake, D. R., Weinheimer, A. J., Woo, J. H., Kim, S. W., Yeo, H., Fried, A. et al. Evaluation of simulated O_3_ production efficiency during the KORUS-AQ campaign: Implications for anthropogenic NO_x_ emissions in Korea. *Elem. Sci. Anth.* (2019).

13. Yang, G. H., Jo, Y. J., Lee, H. J., Song, C. K., and Kim, C. H. Numerical Sensitivity Tests of Volatile Organic Compounds Emission to PM_2.5_ Formation during Heat Wave Period in 2018 in Two Southeast Korean Cities. *Atmosphere* **11**, 331 (2020).

14. Guenther, A., Karl, T., Harley, P., Wiedinmyer, C., Palmer, P. I. and Geron, C. Estimates of global terrestrial isoprene emissions using MEGAN (Model of Emissions of Gases and Aerosols from Nature). *Atmos. Chem. Phys.* **6**(11), 3181–3210 (2006)
